# Comparing Perceived Pain Impact Between Younger and Older Adults With High Impact Chronic Pain: A Cross-Sectional Qualitative and Quantitative Survey

**DOI:** 10.3389/fpain.2022.850713

**Published:** 2022-04-08

**Authors:** Dokyoung S. You, Maisa S. Ziadni, Gabrielle Hettie, Beth D. Darnall, Karon F. Cook, Michael R. Von Korff, Sean C. Mackey

**Affiliations:** ^1^Division of Pain Medicine, Department of Anesthesiology, Perioperative and Pain Medicine, Stanford University School of Medicine, Palo Alto, CA, United States; ^2^Feral Scholars, Broaddus, TX, United States; ^3^Kaiser Permanente Washington Health Research Institute, Seattle, WA, United States

**Keywords:** high impact chronic pain, younger adults, older adults, pain interference, pain impact, CHOIR

## Abstract

High impact chronic pain (HICP) is a recently proposed concept for treatment stratifying patients with chronic pain and monitoring their progress. The goal is to reduce the impact of chronic pain on the individual, their family, and society. The US National Pain Strategy defined HICP as the chronic pain associated with substantial restrictions on participation in work, social, and self-care activities for at least 6 months. To understand the meaning and characteristics of HICP from the younger (<65 years old) and older adults (≥65 years old) with chronic pain, our study examined patients' perceived pain impact between the two age groups. We also characterize the degree of pain impact, assessed with the Patient-Reported Outcomes Measurement Information System (PROMIS) pain interference (PI), between adults and older adults with HICP. We recruited patients at a tertiary pain clinic. The survey included open-ended questions about pain impact, the Graded Chronic Pain Scale-Revised to identify patients' meeting criteria for HICP, and the Patient-Reported Outcomes Measurement Information System (PROMIS^®^) 8-item PI short form (v.8a). A total of 55 younger adults (65.5% women, 72.7% HICP, mean age = 55.0 with *SD* of 16.2) and 28 older adults (53.6% women, 64.3% HICP, mean age = 72.6 with *SD* of 5.4) with chronic pain participated in this study. In response to an open-ended question in which participants were asked to list out the areas of major impact pain, those with HICP in the younger group most commonly listed work, social activity, and basic physical activity (e.g., walking and standing); for those in the older group, basic physical activity, instrumental activity of daily living (e.g., housework, grocery shopping), and participating in social or fun activity for older adults with HICP were the most common. A 2 × 2 ANOVA was conducted using age (younger adults vs. older adults) and HICP classification (HICP vs. No HICP). A statistically significant difference was found in the PROMIS-PI T-scores by HICP status (HICP: *M* = 58.4, *SD* = 6.3; No HICP: *M* = 67.8, *SD* = 6.3), but not by age groups with HICP. In conclusion, perceived pain impacts were qualitatively, but not quantitatively different between younger and older adults with HICP. We discuss limitations and offer recommendations for future research.

## Introduction

Among US adults, about 20.4% are estimated to have chronic pain and 8.0% have chronic pain, which significantly limits daily life or work activities (1). The US National Pain Strategy has introduced a new term, high impact chronic pain (HICP), to identify this latter subgroup with high burden chronic pain and has defined HICP as chronic pain associated with substantial restriction in work, social, and self-care activities for at least 6 months (2). The National Pain Strategy has also called for the development of HICP treatment strategy for patients who face life-limiting chronic pain because such life-limiting chronic pain imposes a significant financial, physical, and emotional burden on patients and also their families and society (2). The estimated total cost for medical treatment, lost productivity, and disability program for chronic pain is $560 billion per year (3). An analysis of the US data from 2003 to 2015 revealed that individual healthcare cost is greater than two-folds for patients with high burden chronic pain ($14,661/year) than patients with low burden chronic pain ($5,979/year) (4) and average US community-dwelling adults in 2015 ($5,141/year) (5). The research findings reveal that HICP is associated with higher pain intensity, more comorbid pain conditions, higher daily opioid dose, worse physical and mental health status, more cognitive impairment, and more healthcare utilization (4, 6–8). These empirical data highlight the complex care needs of the patients with HICP and underscore the important role clinicians may play in both assessing patients for HICP and developing comprehensive care plans to address the unique needs of this population.

The prevalence of chronic pain and HICP increases with age (9). It is observed that about 30.8% of older adults suffer from chronic pain (9) and between 10.7 and 15.8% have HICP (9–11). While chronic pain is more common in older adults (≥65 years of age), relatively few studies on pain have focused on this potentially vulnerable population. So far, the research has found small but significant differences in health status between younger and older adults with chronic pain (12–14). Specifically, older adults with chronic pain generally report worse physical health status and pain-related disability but better mental health and quality of life than younger adults with chronic pain (12, 13). The impact of chronic pain among older adults has been examined by comparing older adults with and without chronic pain. The findings, similar to those from the younger adult literature (15–21), show that older adult chronic pain is associated with poorer physical function (22), sleep (23, 24), mental health (25), cognitive function (26), greater disability (27), and mortality (28, 29). Older adult chronic pain is also associated with accelerated memory decline and increased dementia (30). These findings suggest that chronic pain burden is substantial and potentially of greater consequences for older adults. The research efforts are urgently needed to better understand older adult chronic pain. In the US alone, the older adult population is expected to grow from 17% in 2020 to 23% in 2060 (31).

This study adopted a patient-centered approach to examine the concept of HICP. Based on the National Pain Society definition, HICP is comprised of activity limitations in the three major life domains: work, social, and self-care. To the best of our knowledge, no study has examined whether people with chronic pain endorse such limitations. More specifically, younger and older adults with chronic pain who are at different stages of life may have differing perspectives on major areas of pain impacts. For instance, older adults are more likely to be retired or soon-to-be retired and may not consider work limitation as a significant life impact. Thus, we must consider that HICP could be contextually dependent on potential age-specific factors.

To account for potential age-related variation, we administered an online survey with open-ended questions to younger and older adults with chronic pain to learn about their perceived pain impact and the top three areas of their lives that were most impacted by chronic pain. We also administered two validated measures, the Graded Chronic Pain Scale-Revised (GCPS-R) (32) and Patient-Reported Outcomes Measurement Information System (PROMIS) pain interference (PI) (33) to identify people with and without HICP and the respective degree of pain impact. To analyze these data, we used a mixed-method approach. Our qualitative data analysis identified the top three areas of pain impact. A quantitative analysis of the PROMIS-PI T-scores examined whether the degree of pain impact was significantly different between the following groups. First, we compared major areas of pain impacts and the degree of pain impact between younger and older adults with chronic pain. We further compared perceived pain impacts between the groups with and without HICP regardless of age. Next, we compared pain impacts between the younger and older adult groups with HICP. Finally, we investigated an age by HICP interaction effect by comparing the pain impacts between the two age groups with and without HICP.

We tested the following four hypotheses. First, older adults with chronic pain would differ qualitatively and quantitatively compared to younger adults with chronic pain. Qualitatively, the younger group's responses to the three most impacted areas would be different from the older group's responses. Quantitatively, the older group's PROMIS-PI T-scores would be significantly higher than the younger group's PI T-scores. Second, the impact of chronic pain would be qualitatively and quantitatively different between the groups with and without HICP. Third, when comparing the younger and the older groups with HICP, the three major areas of pain impacts would be qualitatively different and the degree of pain impact would be significantly different. Finally, there might be an age by HICP interaction effect when examining the group differences in pain impacts qualitatively and quantitatively.

## Materials and Methods

The Stanford Institutional Review Board approved the study procedures. Participants were recruited using an open-source data registry at a tertiary pain clinic (https://choir.stanford.edu). Among patients who had the first clinic visit between May and August in 2021, 318 patients agreed to be conducted for a research opportunity. We emailed study invitations two times to these patients. All participants reviewed the consent information prior to completing the current online study survey *via* REDCap, a HIPPA-compliant database. Inclusion criteria were adult patients (at least 18 years of age) with mixed etiology of chronic pain seeking care at a tertiary pain clinic. All responders were aged 18 and older and endorsed at least one chronic pain condition. Although there were no exclusion criteria, the study invitations were sent in English *via* email only. We asked patients to write their responses about pain impacts in a free-text format. As such, patients who did not have fluency in written English, did not have email access, or had trouble in typing their responses on an online survey were naturally excluded. There was no monetary compensation for this study. However, participants were informed that some would have an opportunity to participate in an additional study involving a clinical interview about pain impacts *via* zoom. This additional study would offer a $30 gift card.

### Measures

The survey collected patient's demographic information (e.g., sex, age, education) and pain characteristics (e.g., pain duration, pain intensity ratings).

#### Qualitative Data

We asked patients to describe how their pain impacted their life in free-text format and then pick the top three areas that were most impacted by their chronic pain condition(s). The first question was “*Please tell us how your pain has impacted (changed, limited) your life? (e.g., I can't drive because of pain). Please list all the impacted areas in your life*.” The second question was “*Among all the things listed above, what are the three areas of your life affected by your chronic pain the most?*”.

#### Quantitative Data

The 5-item Graded Chronic Pain Scale-Revised (GCPS-R) (32) was used to classify people as having or not having HICP. The GCPS-R is a validated and widely used measure to differentiate mild, moderate or bothersome, and HICP (Cronbach's α = 0.73–0.89) (32, 34, 35). The first two GCPS-R items are used to classify people with and without HICP. The GCPS-R's criteria for HICP classification are endorsing (1) pain on most days or every day for the past 3 months and (2) pain limiting life or work activities on most days or every day for the past 3 months. A number of three additional items inquire about (3) average pain, (4) degree of PI in the enjoyment of life, and (5) general activities for the past week on a 0–10 scale. The summed score of these three items is used to distinguish mild (< 12) and bothersome pain impact (12 or higher). The GCPS-R has one additional item asking a binary response (yes or no) question, “*Are you not working or unable to work due to pain?*”. This item is not used to assess the pain impact but only assess respondents' work status (32).

The PROMIS-PI 8-item short-form (PROMIS-PI) (33) was administered to assess the degree of pain impact. The PROMIS-PI short form includes items that assess PI with daily activity, housework, social activity, household chores, fun activity, enjoyment of social activity, enjoyment of life, and family life (33, 36). The raw scores were converted to T-scores using the IRT-based web scoring system (https://www.assessmentcenter.net). Higher T-scores indicate greater PI, with a population mean of 50 and a standard deviation of 10. The PROMIS-PI is highly reliable (*r*s = 0.96–0.99) (36).

### A Concurrent Mixed Method Design and Analysis

This cross-sectional study administered a survey to collect the qualitative and quantitative data concurrently (37) about perceived pain impact. With responses to open-ended questions and the validated PROMIS-PI measure, this study conducted the complimentary analysis to better understand perceived pain impact in younger and older adults with and without HICP (37).

#### Qualitative Data Analysis

Using NVIVO 12 software, a coder (DY) conducted a summative content analysis (38). Specifically, initial coding started with the pain impact areas as defined in HICP (work, social activity, and self-care) and listed in the PROMIS-PI item bank (e.g., walking, standing, emotion, cognition, and sleep) (36). Using the deductive approach, responses were coded into one of the existing (34) or new categories as needed. To examine the similarities and differences in perceived pain impacts between the two age groups with and without HICP, we compared the ranking and relative frequency of pain impact areas (39).

#### Quantitative Data Analysis

We used IBM SPSS 26 software for all quantitative data analysis. We conducted an independent *t*-test when comparing the PROMIS-PI T-scores between two groups and a 2 × 2 ANOVA (adults vs. older adults) x (HICP vs. No HICP) when examining the interaction effects between two age groups and HICP status. We calculated effect sizes (partial η^2^) for any significant main or interaction effect. Respective partial η^2^ scores of 0.01, 0.06, and 0.14 would indicate small, medium, and large effect sizes (40).

## Results

### Sample Characteristics

From May 2021 to August 2021, 69 younger adults and 34 older adults attempted to complete the survey (i.e., consented and completed a few initial questions). Of these people who attempted to complete the survey, 55 younger adults (19–64 years) and 28 older adults (66–84 years) completed the survey and provided their responses on perceived pain impact. Therefore, the completion rate was 79.7% in adults, and 82.5% in older adults, χ(1) = 0.102, *p* = 0.750, and we included only those who completed the survey in the current analysis.

Demographic characteristics are summarized in Table 1. The final sample was predominantly female (61.4%), married (62.7%), and highly educated patients (94.0% with at least some college education). As expected, 89.3% of older adults reported their employment status as being retired whereas only 12.7% of younger adults reported being retired. Similarly, the proportion of people endorsing not working due to pain was 25.0% in older adults and 45.5% in younger adults. Disability status was reported in younger adult patients only, with 34.5% of adults reporting being disabled.

**Table 1:** Demographic characteristics.

	**Total** **(*n* = 83)**	**Younger** **adults** **(*n* = 55)**	**Older** **adults** **(*n* = 28)**
	***M*** **(*SD*)**	***M*** **(*SD*)**	***M*** **(*SD*)**
Age (years)	55.0 (16.2)	46.0 (11.8)	72.6 (5.4)
	***n*** **(%)**	***n*** **(%)**	***n*** **(%)**
**Sex**			
Male	30 (36.1)	17 (30.9)	13 (46.4)
Female	51 (61.4)	36 (65.5)	15 (53.6)
Non-binary	2 (2.4)	2 (3.6)	0 (0.0)
**Marital status**			
Single	12 (14.5)	11 (20.0)	1 (3.6)
Married/Living Together	52 (62.7)	33 (60.0)	19 (67.9)
Separated/Divorced	15 (18.1)	11 (20.0)	4 (14.3)
Widowed	4 (4.8)	0 (0.0)	4 (14.3)
**Education**			
High School/GED	5 (6.0)	3 (5.5)	2 (7.1)
Some college	20 (24.1)	15 (27.3)	5 (17.9)
College	34 (41.0)	24 (43.6)	10 (35.7)
Advanced degree	24 (28.9)	13 (23.6)	11 (39.3)
**Employment status**			
Full-time	24 (28.9)	21 (38.2)	3 (10.7)
Part-Time	6 (7.2)	6 (10.9)	0 (0.0)
Retired	32 (38.6)	7 (12.7)	25 (89.3)
Not working	15 (18.1)	15 (27.3)	0 (0.0)
Student	4 (4.8)	4 (7.3)	0 (0.0)
Decline to answer	2 (2.4)	2 (3.6)	0 (0.0)
**Not working due to pain**			
Yes	32 (38.6)	25 (45.5)	7 (25.0)
No	51 (61.4)	30 (54.5)	21 (75.0)
**Disability status**			
Yes	19 (22.9)	19 (34.5)	0 (0.0)
No	64 (77.1)	36 (65.5)	28 (100.0)

### Pain Characteristics

Pain characteristics are summarized in Table 2. The average duration of pain for the sample was 9.6 years (range 0.4–54.0 years), with a mean pain intensity of 6.4 (*SD* = 2.4), and a mean PROMIS-PI T-scores of 65.0 (*SD* = 6.9). More than half endorsed having severe pain among younger adult (56.4%) and older adult patients (57.1%) alike. About 63.6% of younger adults and 42.9% of older adults reported having more than one pain condition. Patients were asked to describe their pain conditions and their responses were mixed with the descriptions of pain locations, diagnosis, and pain etiology and causes (Table 2). Musculoskeletal pain was the most frequently reported pain condition in the total sample (67.5%) and also among younger adults (61.8%) and older adults (78.6%). The second-most endorsed pain condition was any headache or orofacial pain (41.8%) for younger adults and nerve-related pain for older adults (32.1%).

**Table 2:** Pain characteristics.

	**Total** **(*n* = 83)**	**Younger** **adults** **(*n* = 55)**	**Older** **adults** **(*n* = 28))**
	***M*** **(*SD*)**	***M*** **(*SD*)**	***M*** **(*SD*)**
Pain duration (years)	9.6 (10.0)	10.3 (9.4)	8.2 (11.1)
Average pain in the past week	6.4 (2.4)	6.4 (2.6)	6.4 (2.1)
PROMIS-Pain Interference	65.0 (6.9)	65.1 (7.4)	64.7 (5.9)
	***n*** **(%)**	***n*** **(%)**	***n*** **(%)**
**Pain intensity on 0–10 scale**
Mild pain (<4)	11 (13.3)	8 (14.5)	3 (10.7)
Moderate pain (4-6)	25 (30.1)	16 (29.0)	9 (32.1)
Severe pain (>6)	47 (56.6)	31 (56.4)	16 (57.1)
**Pain conditions**
Single	36 (43.7)	20 (36.4)	16 (57.1)
Two or more	47 (56.6)	35 (63.6)	12 (42.9)
Chronic pain conditions^*^
Musculoskeletal	66 (67.5)	34 (61.8)	22 (78.6)
Any headache and orofacial	25 (30.1)	23 (41.8)	2 (7.1)
Nerve-related	16 (19.3)	7 (12.7)	9 (32.1)
Visceral/Pelvic	13 (15.7)	12 (21.8)	1 (3.6)
Inflammation-related	10 (12.0)	9 (16.4)	1 (3.6)
Surgery/Injury-related pain	6 (7.2)	3 (5.5)	3 (10.7)
Cancer-related pain	2 (2.4)	1 (1.8)	1 (3.6)
HICP			
Mild	11 (13.3)	7 (12.7)	4 (14.3)
Moderate/bothersome	14 (16.9)	8 (14.5)	6 (21.4)
High	58 (69.9)	40 (72.7)	18 (64.3)

* *Multiple pain conditions were coded so the sum is >100%*.

### HICP Status

Based on the GCPS-R classification, the total sample included 69.9% with HICP, 16.9% with moderate pain impact, and 13.3% with mild impact pain. The distribution was similar for both younger and older adults. Disproportionally higher prevalence of HICP in our sample is likely a reflection of the selection bias associated with our study sample being drawn from a tertiary pain clinic.

Hypothesis 1: Qualitatively, the younger group's responses to the three most impacted areas would be different from the older group's responses. Quantitatively, the older group's PROMIS-PI T-scores would be significantly higher than the younger group's PI T-scores.

This study compared the three major pain impact areas between the younger and older groups regardless of HICP status (Table 3). More than 50% of patients described the impact of pain on their life as their basic physical activities (e.g., walking, sitting, standing, lifting, bending, grabbing, pulling, using stairs, any physical activity). Among the basic physical activities, the most frequently endorsed limited activity was walking, followed by any physical activity, and sitting for both groups. The second-most frequently endorsed pain impact was an instrumental activity of daily living (ADL). The most frequently endorsed pain limiting instrumental ADL was housework, followed by driving or riding a car, and grocery shopping for both adult and older adult groups. The third-most frequently endorsed pain impact area was an exercise for the older adult group and work for the younger adult group. As expected, pain impact on work was less frequently endorsed (ranked ninth) among older adults with chronic pain. For younger adults, the other major areas impacted by chronic pain were in the order of exercise, social activity with family members or non-family members, mental health (e.g., depression, anxiety, worry, and lost motivation for activity), sleep, self-care, cognitive function (e.g., concentration difficulty, memory issue, and mental sluggish), fun activity, fatigue, intimate relationship, lying in bed all the day, increased cognitive burden for activity planning and being more cautious in engaging activities, and relationship with family (e.g., not fully present in interacting with children). The other major pain impact areas for older adults were social activity, sleep, fun activity, self-care, increased cognitive burden, work, mental health, lying in bed all day, fatigue, intimate relationship, and relationship with the family.

**Table 3:** Comparing the three major areas of pain impact between younger and older adults with chronic pain.

	**Total** ***n* = 83** **(%)**	**Younger** **adults** ***n*** **= 55** **(%)**	**Older** **adults** ***n*** **= 28** **(%)**
1. Basic physical activity	53.0	54.5	50.0
2. Instrumental ADLs	48.2	49.1	46.4
3. Exercise	32.5	34.5	28.6
4. Social activity	28.9	30.9	25.0
5. Work	28.9	40.0	7.1
6. Sleep	21.7	21.8	21.4
7. Mental health (depression, anxiety, worry)	18.1	23.6	7.1
8. Self-care	16.9	16.4	17.9
9. Fun activity (Leisure, Hobby)	15.7	12.7	21.4
10. Cognitive function	10.8	16.4	0.0
11. Fatigue/Low energy	8.4	10.9	3.6
12. Lying in bed all day and do nothing	6.0	5.5	7.1
13. Intimate relationship and sexual life	6.0	7.3	3.6
14. Cognitive burden for activity planning and engagement	6.0	3.6	10.7
15. Relationship with family	2.4	3.6	0.0
	*M* (*SD*)	*M* (*SD*)	*M* (*SD*)
PROMIS-PI T-scores	65.0 (6.9)	65.1 (7.4)	64.7 (5.9)

*The darker red, the more frequently endorsed pain impact*.

We conducted an independent *t*-test to compare the PROMIS-PI T-scores between the younger and older groups to examine the quantitative difference in pain impact. The result revealed no significant difference between the two age groups, *t*(81) = 0.245, *p* = 0.087.

Taken together, these results partially support Hypothesis 1. As hypothesized, there were differences in the major areas of pain impact between the two age groups. Specifically, pain impact on work, mental health, and cognitive function were more frequently reported by younger adults with chronic pain, and the pain impact on fun activities and the cognitive burden was more frequently reported by older adults with chronic pain. Contrary to Hypothesis 1, more than half of patients in both age groups described the areas impacted by chronic pain as the basic physical activity and instrumental ADLs. Furthermore, the PROMIS-PI T-scores were not significantly higher in older adults with chronic pain than younger adults with chronic pain.

Hypothesis 2: The impact of chronic pain would be qualitatively and quantitatively different between the groups with and without HICP.

We extracted participant selections of three major pain impacts and compared their responses between the groups with and without HICP regardless of age (Table 4). Of the HICP group, basic physical activity, instrumental ADLs, and work were the three most frequently endorsed major pain impact areas. It should be noted that pain interfering work, social activity, and self-care activity were not specific to the group with HICP. The other major pain impact endorsed by the group with HICP, but not by the group without HICP, was lying in bed all the day and doing nothing, suggesting HICP as being associated with substantial restrictions in life activities. Additionally, pain limiting exercise and fun activities were more endorsed by the group without HICP.

**Table 4:** Comparing the three major areas of pain impact between adults and older adults with and without HICP.

	**No HICP** ***n*** **= 25** **(%)**	**HICP** ***n*** **= 58** **(%)**
1. Basic physical activity	44.0	56.9
2. Instrumental activity of daily living	40.0	51.7
3. Work	24.0	31.0
4. Social activity	28.0	29.3
5. Exercise	44.0	27.6
6. Sleep	16.0	24.1
7. Mental health	20.0	17.2
8. Self-care	20.0	15.5
9. Fun activity	24.0	12.1
10. Cognitive function	12.0	10.3
11. Fatigue/Low Energy	8.0	8.6
12. Lying in bed all day and do nothing	0.0	8.6
13. Intimate relationship and sexual life	4.0	6.9
14. Cognitive burden for activity planning and engagement	4.0	6.9
15. Relationship with family	4.0	1.7
	***M*** **(*****SD*****)**	***M*** **(*****SD*****)**
PROMIS-PI T-scores	58.5 (6.3)	67.8 (5.1)

*The darker red, the more frequently endorsed pain impact*.

The result of an independent *t*-test indicated a significant difference between the groups with and without HICP, *t*(81) = 7.049, *p* < 0.00001. The mean of the PROMIS-PI T-scores was 58.5 (*SD* = 6.3) for the group without HICP and 67.8 (*SD* = 5.1) for the group with HICP, which is a large difference (Cohen's *d* = 1.62).

Taken together, our results support Hypothesis 2. The groups with and without HICP were qualitatively different in the major impact areas of chronic pain and quantitatively different in the degree of pain impact.

Hypothesis 3: When comparing the younger and the older groups with HICP, the three major areas of pain impacts would be qualitatively different, and the degree of pain impact would be significantly different.

We extracted participants' selections of three major pain impacts and compared their responses between the younger and older groups with HICP (Table 5, right two columns). Of the younger adult group with HICP, basic physical activity, instrumental ADLs, and work were the three most frequently endorsed major areas of pain impact. The most frequently endorsed major pain impacts of the older adult group with HICP were basic physical activity and instrumental ADLs. Next, the older adult group with HICP reported social activity, exercise, sleep, and fun activity as the primary pain impact areas. The impact of pain on work was ranked seventh. These results suggest that the three major areas of pain impact may be qualitatively different between younger and older adults with HICP. The result of an independent *t*-test indicated no significant difference between the younger and older groups with HICP, *t*(56) = 0.918, *p* = 0.918.

**Table 5:** Comparing the three major areas of pain impact between adults and older adults with and without HICP.

	**No HICP**		**HICP**	
	**Younger** ***n*** **= 15** **(%)**	**Older** ***n*** **= 10** **(%)**	**Younger** ***n*** **= 40** **(%)**	**Older** ***n*** **= 15** **(%)**
1: Basic physical activity	46.7	40.0	57.5	55.6
2: Instrumental ADLs	40.0	40.0	52.5	50.0
3: Work	40.0	0.0	40.0	11.1
4: Social activity	20.0	40.0	35.0	16.7
5: Exercise	40.0	50.0	32.5	16.7
6: Sleep	6.7	30.0	27.5	16.7
7: Mental Health	26.7	10.0	22.5	5.6
8: Self-care	13.3	30.0	17.5	11.1
9: Cognitive function	20.0	0.0	15.0	0.0
10: Fatigue/Low Energy	13.3	0.0	10.0	5.6
11: Fun Activity	20.0	30.0	10.0	16.7
12: Lying in bed all day and do nothing	0.0	0.0	7.5	11.1
13: Intimate relationship and sex life	6.7	0.0	7.5	5.6
14: Cognitive burden for activity planning and engagement	0.0	10.0	5.0	11.1
15: Relationship with family	6.7	0.0	2.5	0.0
	***M*** **(*****SD*****)**	***M*** **(*****SD*****)**	***M*** **(*****SD*****)**	***M*** **(*****SD*****)**
PROMIS-PI T-scores	57.9 (7.6)	59.5 (3.7)	67.8 (5.3)	67.8 (5.1)

*The darker red, the more frequently endorsed pain impact*.

Taken together, the Hypothesis 3 was partially supported. The younger and older groups with HICP were qualitatively different in the major impacts of chronic pain, but not quantitatively different in the degree of pain impact.

Hypothesis 4: There might be an age by HICP interaction effect when examining the group differences in pain impacts qualitatively and quantitatively.

Table 5 shows the differences in major areas of pain impact between the two age groups with and without HICP. The major pain impacts endorsed by younger adults with HICP, but not by younger adults without HICP, were lying in bed all the day. The major pain impacts endorsed by older adults with HICP, but not by older adults without HICP, were work, fatigue, lying in bed all the day, and intimate relationship. Of the older adult groups, pain limiting self-care was only reported by older adults without HICP.

**Figure 1:**
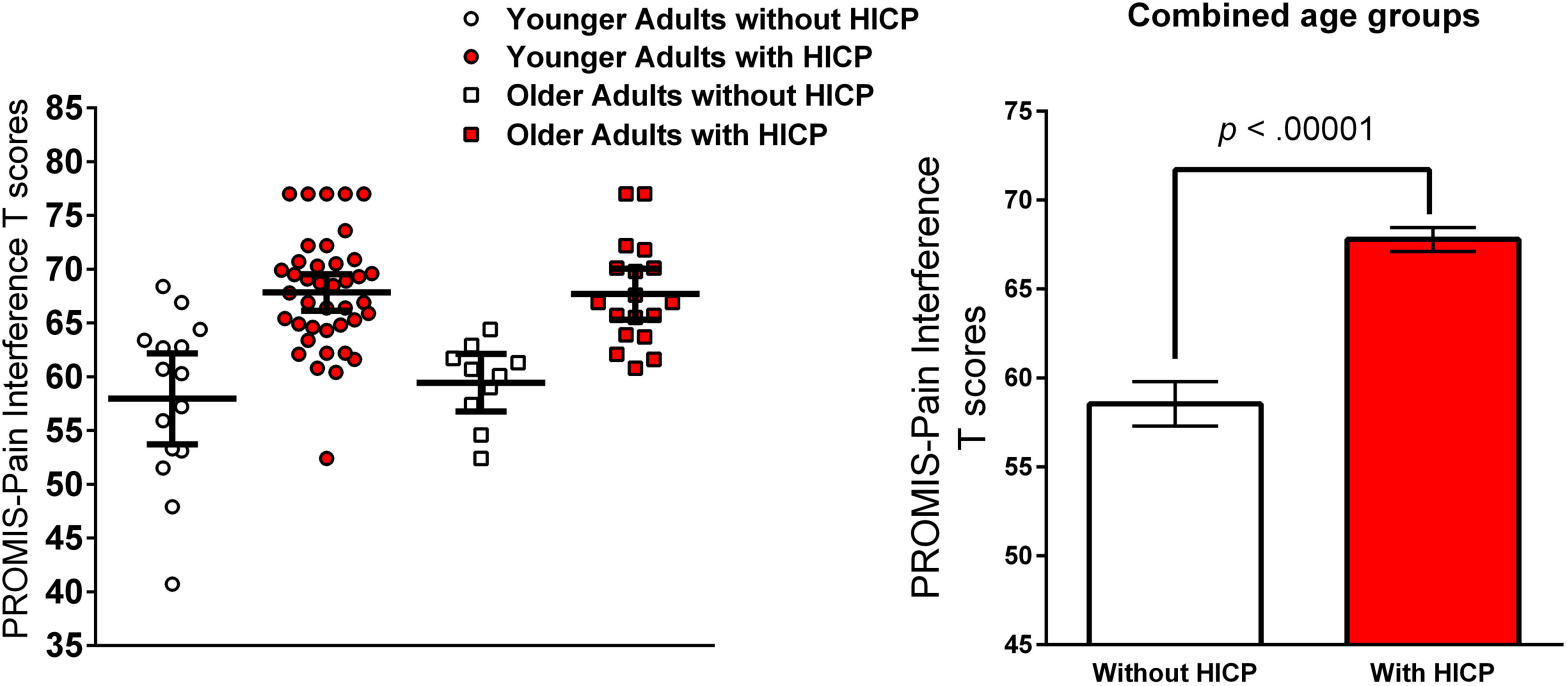
PROMIS-PI T scores younger and older adults with and without HICP (left) and error bars are 95% Confidence Interval. Comparison of PROMIS-PI T scores between the group with and without HICP (right) and error bars are SEMs.

A 2 × 2 ANOVA (younger adults vs. older adults) × (HICP vs. No HICP) was conducted to examine whether the PROMIS-PI T-scores were significantly different between the two age groups with and without HICP. The results indicated no significant age by HICP status interaction, *F*_(1, 79)_ = 0.361, *p* = 0.361 and no significant main effect of age, *F*_(1, 79)_ = 0.241, *p* = 0.625, but a significant main effect of HICP status, and *F*_(1, 79)_ = 43.412, *p* < 0.00001, partial η^2^ = 0.36 (see Figure 1, left). As also being reported previously in Hypotheses 2 and 3, both age groups with HICP reported significantly higher PROMIS-PI T-scores than the groups without HICP (see Figure 1, right).

The above results partially support Hypothesis 4. The major pain impact areas were qualitatively different between the two age groups with and without HICP, which suggests age by HICP interaction effect. In quantitative analysis, older adults with HICP reported significantly higher PI than older adults without HICP, but they did not report significantly higher PI than younger adults with HICP, which suggests no significant age by HICP interaction effect.

## Discussion

This study used a patient-centered approach to examine the meaning and characteristics of HICP among younger adults and older adults with chronic pain because the patients' perception of HICP had not been examined. Furthermore, HICP might have a different meaning when applied to older adults with chronic pain. Older adults are usually retired or soon-to-be retired, placing less importance on the pain impact on work than younger adults with chronic pain. Therefore, we administered open-ended questions to allow patients to identify their own perceived pain impacts without providing guidance or limited options. We analyzed qualitative data of patients' responses to the open-ended questions and quantitative data of the PROMIS PI T-scores. The following four were the major findings.

First, we examined whether the major areas of pain impact would be different between younger adults (aged 18–64) and older adults (≥65 years) with chronic pain. Our results suggested that there were similarities and differences in major pain impacts between the two age groups. Both younger and older adult patients reported most frequently that their pain impacted basic physical activity (e.g., walking, sitting, and standing) and instrumental ADLs (e.g., housework, driving, and grocery shopping). As expected, we found the differences in pain impact on work between the two age groups. About 40% of younger adults with chronic pain reported the pain impact on their work whereas only 7% of older adults with chronic pain did. These results were expected in light of reported US average retirement ages of 64 years for men and 62 years for women (41). In our sample, 56.4% of younger adults reported their current employment status as working or being a student, and only 10.7% of older adults reported currently working. Another age-related difference was observed in mental health, with younger adults with chronic pain more frequently reporting the pain impact on mental health. This result is consistent with other studies showing that younger adults with chronic pain reported higher levels of depression, anxiety, negative mood, and fear of movement than older adults with chronic pain (12–14, 42, 43). A potential reason for less pain impact on mental health in older adults might be due to greater general resilience (44, 45) or using more adaptive pain coping strategies in older adults (46, 47), but this age-related change in pain coping strategy remains to be elucidated.

Second, we examined whether the top three major pain impacts would differ between the groups with and without HICP regardless of age. As hypothesized, the group with HICP reported qualitatively and quantitatively different pain impacts than those without HICP. The two major areas of pain impact were basic physical activity and instrumental ADLs for both groups, but the third-most frequently impacted areas were work for the group with HICP and exercise for the group without HICP. Additionally, lying in bed all the day and doing nothing was endorsed only by the group with HICP. Furthermore, the PROMIS-PI T-scores were significantly higher in the group with HICP than the group with HICP, which was a large difference. Both qualitative and quantitative analyses indicated HICP as being associated with substantial limitations in life activity.

Next, we compared the three major areas of pain impact between the younger and older adults with HICP and their responses with the expert definition of HICP. In our younger adult HICP group, basic physical activity, instrumental ADLs, and work were identified as the top three major pain impacts, with social activity ranked fourth and self-care ranked eighth. In our older adults with HICP group, basic physical activity and instrumental ADL were the most frequently impacted areas. Social activity, exercise, sleep, and fun activity were ranked third. Furthermore, work was ranked seventh. Therefore, the meaning and characteristics of HICP appeared to be different between younger adults and older adults with chronic pain.

Finally, this study examined whether there might be an age by HICP status interaction effect in pain impact. Our qualitative analysis on younger adult patients' responses revealed that the three major life domains (basic physical activity, instrumental ADLs, and work) were not specific to patients with HICP. Lying in bed all the day was the only pain impact area specific to HICP in the younger adult group. For older adults, pain impact areas that are specific to HICP were work, fatigue, lying in bed all the day, and intimate relationship. Therefore, the major areas of pain impact appeared to be different between the two age groups with and without HICP. When quantitatively evaluating the age by HICP interaction effect, our results indicated no significant age by HICP status interaction effect. We found a significant difference in PROMIS-PI T-scores between older adults with and without HICP, but no significant difference between younger and older adults with HICP. The PROMIS-PI assesses the pain impact for the past week, and the current expert definition of HICP refers to the pain impact for an extended period (6 months or more). Although the PROMIS-PI T-scores reflect the pain impact only in the recent 7 days, our finding suggests that the degree of chronic pain impact may not be significantly different between younger and older adults with HICP.

Concerning HICP assessment, we proposed that the PROMIS-PI may be a stronger candidate measure than the GCPS-R. While the 5-item GCPS-R (32) is widely used, only two items are used to identify people with HICP. One item assesses the frequency of pain and the other item assesses the frequency of life- or work-limiting pain in the past 3 months. Compared to the perceived pain impacts endorsed by our patients, the GCPS-R appears to be a screening tool. The PROMIS-PI assesses the degree of pain impact on multiple life domains, such as physical activity, social activity, recreational activity, sleep, emotion, cognitive function, and enjoyment in life, all of which have been endorsed by our patients (36). This is somewhat expected because the PROMIS items have been developed from the literature reviews and input from patients with chronic pain (36). Furthermore, the PROMIS uses an IRT approach, which calculates the severity parameter of each item and allows to compare seemingly different pain impact items on the single construct of pain impact or interference (θ, mean of 0, *SD* of 1.0). For example, a response of “always” on an item about pain interfering sitting more than 10 min is associated with θ-values of 2.971 and response of “very much” on an item about pain interfering concentration is associated with a θ-value of 2.329 (36), with higher θ scores indicating greater pain impact. Based on the patient's responses on the PROMIS items, each patient will have a θ estimation score with a standard error of that estimated score. The θ scores can be converted to T-score (*M* = 50, *SD* = 10). Overall, the PROMIS-PI items appear to match well with our patients' perceived pain impact and may be a stronger candidate measure to assess HICP. More research is needed to assess the extended nature (i.e., duration) of HICP with the PROMIS PI and to empirically identify the cutoff scores for HICP.

Our findings have implications in age-related considerations for pain care. For younger adults with chronic pain, mental health and work were endorsed more frequently as the major pain impact areas. To reduce the pain impact on emotion and work, clinicians should consider pain psychology (e.g., cognitive behavioral therapy and acceptance and commitment therapy) or multidisciplinary programs, which were found to be effective in reducing emotional distress, returning to work, and reducing the number of sick leave days (48–53). Compared to younger adults with chronic pain, older adults with chronic pain endorsed more frequently that the pain interfered with fun activity and increased cognitive burden in activity planning and engagement. Indeed, an interview study revealed that older adults considered having fun, enjoying life, and having a sense of humor as successful aging (54). Clinicians should assess unmet desire for having fun in life and help to engage in a fun activity (e.g., scheduling fun/pleasant activity) (55). Additionally, clinicians should consider occupational therapy to reduce the cognitive burden for activity planning and engagement as occupational therapy would help people build confidence in daily planning and lifestyle modification (56). Although not frequent, older adults with HICP endorsed pain interfering with intimate relationships and sexual life. It was observed that sexual life was an essential factor for the quality of life in older adults (57). A study on community-dwelling US older adults revealed that 50.9% of men and 30.8% of women were sexually active (58). Older adults were interested in conversing about sexual life with physicians (58), and they wanted physicians to initiate the discussion (59). Regardless of age groups, the major pain impact areas were basic physical activity, instrumental ADLs, exercise, social activity, and sleep, which highlights the need for multidisciplinary pain care for both younger and older adults with chronic pain. The biopsychosocial approach should also be considered in improving the quality of life of people who are living with chronic pain. A study found that pain catastrophizing, not pain intensity, was associated with overall health, mental health, and social function (60). Brief treatments, such as a single-session pain psychology class (61) and pain neuroscience education (62), have been demonstrated to be effective in reducing pain catastrophizing.

Our findings should be interpreted with several important limitations. We had a relatively small sample, with lower representation from older adults (*n* = 28). As such, comparisons between older adults with and without HIPC involved small cell sizes and replication is needed with the larger sample size. Next, the study was enriched for more symptomatic patients and greater prevalence of HICP as the sample was drawn from a tertiary pain clinic; as such, results may not generalize outside of this patient population. The sample was predominantly women, married, highly educated, and less disabled. This study did not assess race or ethnicity, but our clinic sample was predominantly non-Hispanic/White (63–67%), followed by Hispanic/Latino (10%), Asian (6–7%), and African American/Black (3%) (63, 64). Our sample was also limited to English speakers and those with email access. Therefore, results may not generalize to patients with greater diversity, race or ethnicity, education, and ability status. Broadly, future research on this topic should include diverse and large sample sizes. Finally, this study compared pain impact areas between groups based on the chronological age, but some evidence suggested perceived age (i.e., perceived younger or older than one's chronological age) as being a significant predictor for pain impact (65). The future study should investigate perceived pain impact areas between the groups based on the perceived age.

With these limitations acknowledged, this study contributes to the current literature for the following reasons. It is the first study to examine the characteristics of HICP with the inputs from patients with chronic pain. We analyzed qualitative and quantitative data to better understand perceived pain impacts, and our qualitative data included open-ended questions to obtain unguided responses of patients with HICP. Although the study asked about the pain impacted on “*life*,” more than 50% of patients described their perceived pain impact on *basic physical activities*, such as walking, sitting, and standing, and a few described its impact on different symptoms, such as depression, anxiety, fatigue, sleep, and difficulty concentrating. It should also be noted that the current definition of HICP applies to the pain impact on major life *activities* (i.e., work, social, and self-care), rather than its impacts on additional symptoms or functions (e.g., sleep, emotions, fatigue, and cognitive function). Additional studies with clinical interviews are needed to understand and characterize the patients' perceived pain impact deeply. We also found *increased cognitive burden for activity planning and taking extra-caution for activity engagement* associated with HICP. The pain impact on cognitive burden has not been previously identified and is not included in the PROMIS-PI item bank. The relationship between HICP and the increased cognitive burden is largely unknown. One study on adolescents with chronic pain reports increased cognitive burden for hiding pain symptoms to avoid social judgment, avoid being a social burden, and be treated normally (66), which is also worth exploring in adults with chronic pain. Notably, this study did not conduct a priori power analysis and had a small sample size, but other studies with a large sample size consistently found no significant difference in the PROMIS-PI scores between younger and older adults with chronic pain (14, 67, 68). This study noted the effect size, which would be useful for a priori sample size calculation in the future study comparing the groups with and without HICP.

The National Pain Strategy introduced a new concept of HICP for better classification, treatment, and monitoring of people with high burden chronic pain. These findings suggested that the meaning and characteristics of HICP would be qualitatively, but not quantitatively, different between younger adults and older adults with chronic pain. Therefore, older adults with HICP might have different healthcare needs. These findings may have significant clinical implications. First, validated measures, such as the PROMIS-PI, may be used to assess the degree of chronic pain impact for both younger and older adults. Second, when using validated measures, one cutoff score may be established to screen for HICP among younger and older adults with chronic pain. Finally, to develop a treatment strategy for HICP, clinicians may need to assess age-relevant areas of pain impacts and the unique healthcare needs of younger and older adult populations. The current growing research effort to develop an effective treatment strategy for HICP should also consider this age-related difference in perceived pain impacts and pain care needs. We plan to conduct additional studies to further understand the meaning of HICP from the patients living with chronic pain and to appropriately characterize the HICP of younger and older adults.

## Data Availability Statement

The raw data supporting the conclusions of this article will be made available by the authors, without undue reservation.

## Ethics Statement

The studies involving human participants were reviewed and approved by Stanford. The patients/participants provided their written informed consent to participate in this study.

## Author Contributions

DY, KC, MV, and SM contributed to conception and design of the study. GH organized the database. DY performed the statistical analysis and wrote the first draft of the manuscript. MZ, GH, BD, KC, MV, and SM wrote sections of the manuscript. All authors contributed to manuscript revision, read, and approved the submitted version.

## Funding

DY (K23DA048972), MZ (K23DA047473), and BD (K24DA053564) received funding from the NIH National Institute on Drug Abuse. SM (K24NS126781, R61NS11865) received funding from the NIH National Institute of Neurological Disorders and Stroke.

## Conflict of Interest

The authors declare that the research was conducted in the absence of any commercial or financial relationships that could be construed as a potential conflict of interest.

## Publisher's Note

All claims expressed in this article are solely those of the authors and do not necessarily represent those of their affiliated organizations, or those of the publisher, the editors and the reviewers. Any product that may be evaluated in this article, or claim that may be made by its manufacturer, is not guaranteed or endorsed by the publisher.

## References

[1] Dahlhamer J Lucas J Zelaya C Nahin R Mackey S DeBar L. Prevalence of chronic pain and high-impact chronic pain among adults—United States, 2016. Morbid Mortal Wkly Rep. (2018) 67:1001–6. 10.15585/mmwr.mm6736a2 30212442 PMC6146950

[2] Interagency Pain Research Coordinating Committee. A comprehensive population healthlevel strategy for pain: National pain strategy. Washington, DC (2016). Available at: https://www.iprcc.nih.gov/sites/default/files/documents/NationalPainStrategy_508C.pdf

[3] Simon LS. Relieving pain in America: A blueprint for transforming prevention, care, education, and research. J Pain Palliat Care Pharmacother. (2012) 26:197–8. 10.3109/15360288.2012.67847327136641

[4] Herman PM Broten N Lavelle TA Sorbero ME Coulter ID. Health care costs and opioid use associated with high-impact chronic spinal pain in the United States. Spine. (2019) 44:1154–61. 10.1097/BRS.0000000000003033 31373999 PMC6758926

[5] Health Care Cost Institute. 2015 Health Care Cost and Utilization Report. Washington DC (2016).

[6] Pitcher MH Von Korff M Bushnell MC Porter L. Prevalence and profile of high-impact chronic pain in the United States. J Pain. (2019) 20:146–60. 10.1016/j.jpain.2018.07.006 30096445 PMC8822465

[7] Almalki MT BinBaz SS Alamri SS Alghamdi HH El-Kabbani AO Al Mulhem AA. Prevalence of chronic pain and high-impact chronic pain in Saudi Arabia. Saudi Med J. (2019) 40:1256–66. 10.15537/smj.2019.12.24690 31828278 PMC6969620

[8] Von Korff M Scher AI Helmick C Carter-Pokras O Dodick DW Goulet J. United States national pain strategy for population research: concepts, definitions, and pilot data. J Pain. (2016) 17:1068–80. 10.1016/j.jpain.2016.06.009 27377620

[9] Zelaya CE Dahlhamer JM Lucas JW Connor EM. Chronic pain and high-impact chronic pain among US adults, 2019. NCHS Data Brief. (2020) 390:1–8. Available at: https://stacks.cdc.gov/view/cdc/97308 33151145

[10] Johannes CB Le TK Zhou X Johnston JA Dworkin RH. The prevalence of chronic pain in United States adults: results of an Internet-based survey. J Pain. (2010) 11:1230–9. 10.1016/j.jpain.2010.07.002 20797916

[11] Larsson C Hansson EE Sundquist K Jakobsson U. Chronic pain in older adults: prevalence, incidence, and risk factors. Scand J Rheumatol. (2017) 46:317–25. 10.1080/03009742.2016.1218543 27885914

[12] Manogharan S Kongsted A Ferreira M Hancock M. Do older adults with chronic low back pain differ from younger adults in regards to baseline characteristics and prognosis? Eur J Pain. (2017) 21:866–73. 10.1002/ejp.989 28295893

[13] Rustøen T Wahl AK Hanestad BR Lerdal A Paul S Miaskowski C. Age and the experience of chronic pain: differences in health and quality of life among younger, middle-aged, and older adults. Clin J pain. (2005) 21:513–23. 10.1097/01.ajp.0000146217.31780.ef 16215337

[14] Ziadni MS You DS Johnson L Lumley MA Darnall BD. Emotions matter: The role of emotional approach coping in chronic pain. Eur J Pain. (2020) 24:1775–84. 10.1002/ejp.1625 32603553 PMC7923247

[15] Antonucci LA Taurino A Laera D Taurisano P Losole J Lutricuso S. An Ensemble of psychological and physical health indices discriminates between individuals with chronic pain and healthy controls with high reliability: a machine learning study. Pain Ther. (2020) 9:601–14. 10.1007/s40122-020-00191-3 32880867 PMC7648771

[16] Burke AL Mathias JL Denson LA. Psychological functioning of people living with chronic pain: A meta-analytic review. Br J Clin Psychol. (2015) 54:345–60. 10.1111/bjc.12078 25772553

[17] Call-Schmidt TA Richardson SJ. Prevalence of sleep disturbance and its relationship to pain in adults with chronic pain. Pain Manage Nurs. (2003) 4:124–33. 10.1016/S1524-9042(02)54212-0 14566710

[18] Nadar MS Jasem Z Manee FS. The cognitive functions in adults with chronic pain: a comparative study. Pain Res Manage. (2016) 2016:5719380. 10.1155/2016/5719380 28127233 PMC5227177

[19] Baker KS Gibson S Georgiou-Karistianis N Roth RM Giummarra MJ. Everyday executive functioning in chronic pain: specific deficits in working memory and emotion control, predicted by mood, medications, and pain interference. Clin J Pain. (2016) 32:673–80. 10.1097/AJP.0000000000000313 26626294

[20] Macfarlane GJ Barnish MS Jones GT. Persons with chronic widespread pain experience excess mortality: longitudinal results from UK Biobank and meta-analysis. Ann Rheum Dis. (2017) 76:1815–22. 10.1136/annrheumdis-2017-211476 28733474

[21] Fine PG. Long-term consequences of chronic pain: mounting evidence for pain as a neurological disease and parallels with other chronic disease states. Pain Med. (2011) 12:996–1004. 10.1111/j.1526-4637.2011.01187.x 21752179

[22] Makino K Lee S Bae S Jung S Shinkai Y Chiba I. Pain characteristics and incidence of functional disability among community-dwelling older adults. PLoS ONE. (2019) 14:e0215467. 10.1371/journal.pone.0215467 30986257 PMC6464340

[23] Blågestad T Pallesen S Lunde L-H Sivertsen B Nordhus I-H Grønli J. Sleep in older chronic pain patients: a comparative polysomnographic study. Clin J Pain. (2012) 28:277–83. 10.1097/AJP.0b013e3182313899 22330128

[24] Lunde L-H Pallesen S Krangnes L Nordhus IH. Characteristics of sleep in older persons with chronic pain: a study based on actigraphy and self-reporting. Clin J Pain. (2010) 26:132–7. 10.1097/AJP.0b013e3181b61923 20090440

[25] Eggermont LH Penninx BW Jones RN Leveille SG. Depressive symptoms, chronic pain, and falls in older community-dwelling adults: the MOBILIZE Boston study. J Am Geriatr Soc. (2012) 60:230–7. 10.1111/j.1532-5415.2011.03829.x 22283141 PMC3288166

[26] Van Der Leeuw G Eggermont LH Shi L Milberg WP Gross AL Hausdorff JM. Pain and cognitive function among older adults living in the community. J Gerontol Ser A BiomedSci Med Sci. (2016) 71:398–405. 10.1093/gerona/glv166 26433218 PMC5013972

[27] Hairi NN Cumming RG Blyth FM Naganathan V. Chronic pain, impact of pain and pain severity with physical disability in older people—Is there a gender difference? Maturitas. (2013) 74:68–73. 10.1016/j.maturitas.2012.10.001 23103063

[28] Smith D Wilkie R Uthman O Jordan JL McBeth J. Chronic pain and mortality: a systematic review. PLoS ONE. (2014) 9:e99048. 10.1371/journal.pone.0099048 24901358 PMC4047043

[29] Smith D Wilkie R Croft P McBeth J. Pain and mortality in older adults: the influence of pain phenotype. Arthritis Care Res. (2018) 70:236–43. 10.1002/acr.23268 28589671

[30] Whitlock EL Diaz-Ramirez LG Glymour MM Boscardin WJ Covinsky KE Smith AK. Association between persistent pain and memory decline and dementia in a longitudinal cohort of elders. JAMA Intern Med. (2017) 177:1146–53. 10.1001/jamainternmed.2017.1622 28586818 PMC5588896

[31] Vespa J Armstrong DM Medina L. Demographic turning points for the United States: population projections for 2020 to 2060: US Department of Commerce, Economics and Statistics Administration, US (2018).

[32] Von Korff M DeBar LL Krebs EE Kerns RD Deyo RA Keefe FJ. Graded chronic pain scale revised: mild, bothersome, and high impact chronic pain. Pain. (2020) 161:651. 10.1097/j.pain.0000000000001758 31764390 PMC7097879

[33] Kean J Monahan P Kroenke K Wu J Yu Z Stump T. Comparative responsiveness of the PROMIS pain interference short forms, brief pain inventory, PEG, and SF-36 bodily pain subscale. Med Care. (2016) 54:414–21. 10.1097/MLR.0000000000000497 26807536 PMC4792763

[34] Krebs EE Lorenz KA Bair MJ Damush TM Wu J Sutherland JM. Development and initial validation of the PEG, a three-item scale assessing pain intensity and interference. J Gen Intern Med. (2009) 24:733–8. 10.1007/s11606-009-0981-1 19418100 PMC2686775

[35] Sharma S Kallen MA Ohrbach R. Graded chronic pain scale: validation of 1-month reference frame. Clin J Pain. (2022) 38:119–31. 10.1097/AJP.0000000000001005 34803153 PMC8727576

[36] Amtmann D Cook KF Jensen MP Chen W-H Choi S Revicki D. Development of a PROMIS item bank to measure pain interference. Pain. (2010) 150:173–82. 10.1016/j.pain.2010.04.025 20554116 PMC2916053

[37] Small ML. How to conduct a mixed methods study: recent trends in a rapidly growing literature. Annu Rev Sociol. (2011) 37:57–86. 10.1146/annurev.soc.012809.102657

[38] Hsieh H-F Shannon SE. Three approaches to qualitative content analysis. Qual Health Res. (2005) 15:1277–88. 10.1177/1049732305276687 16204405

[39] Erickson F. Qualitative Research Methods for Science Education. Second International Handbook of Science Education. Berlin: Springer (2012). p. 1451–69. 10.1007/978-1-4020-9041-7_93

[40] Cohen J. Statistical Power Analysis for the Behavioral Sciences. UK: Routledge (2013). 10.4324/9780203771587

[41] Munnell AH. The average retirement age. Issue in Brief 15–4. Chestnut Hill, MA: Center for Retirement Research at Boston College, (2015). Available online at: http://hdl.handle.net/2345/bc-ir:103786 (accessed March 23, 2022)

[42] Krok JL Baker TA McMillan SC. Age differences in the presence of pain and psychological distress in younger and older cancer patients. J Hospice Palliative Nurs. (2013) 15:107–13. 10.1097/NJH.0b013e31826bfb63

[43] Riley JL III Wade JB Robinson ME Price DD. The stages of pain processing across the adult lifespan. J Pain. (2000) 1:162–70. 10.1016/S1526-5900(00)90101-931513849

[44] Löckenhoff CE Carstensen LL. Socioemotional selectivity theory, aging, and health: The increasingly delicate balance between regulating emotions and making tough choices. J Pers. (2004) 72:1395–424. 10.1111/j.1467-6494.2004.00301.x 15509287

[45] Gooding P Hurst A Johnson J Tarrier N. Psychological resilience in young and older adults. Int J Geriatr Psychiatry. (2012) 27:262–70. 10.1002/gps.2712 21472780

[46] Molton I Jensen MP Ehde DM Carter GT Kraft G Cardenas DD. Coping with chronic pain among younger, middle-aged, and older adults living with neurological injury and disease. J Aging Health. (2008) 20:972–96. 10.1177/0898264308324680 18791184 PMC2716650

[47] Lachapelle DL Hadjistavropoulos T. Age-related differences among adults coping with pain: evaluation of a developmental life-context model. Can J Behav Sci. (2005) 37:123–37. 10.1037/h0087250

[48] Marhold C Linton SJ Melin L. A cognitive–behavioral return-to-work program: effects on pain patients with a history of long-term versus short-term sick leave. Pain. (2001) 91:155–63. 10.1016/S0304-3959(00)00431-0 11240088

[49] Nazarov S Manuwald U Leonardi M Silvaggi F Foucaud J Lamore K. Chronic diseases and employment: which interventions support the maintenance of work and return to work among workers with chronic illnesses? A systematic review. Int J Environ Res Public Health. (2019) 16:1864. 10.3390/ijerph16101864 31137817 PMC6572561

[50] Berglund E Anderzén I Andersén Å Carlsson L Gustavsson C Wallman T. Multidisciplinary intervention and acceptance and commitment therapy for return-to-work and increased employability among patients with mental illness and/or chronic pain: a randomized controlled trial. Int J Environ Res Public Health. (2018) 15:2424. 10.3390/ijerph15112424 30384498 PMC6266920

[51] Aasdahl L Pape K Vasseljen O Johnsen R Gismervik S Halsteinli V. Effect of inpatient multicomponent occupational rehabilitation versus less comprehensive outpatient rehabilitation on sickness absence in persons with musculoskeletal-or mental health disorders: a randomized clinical trial. J Occup Rehabil. (2018) 28:170–9. 10.1007/s10926-017-9708-z 28401441 PMC5820389

[52] Bennett R Nelson D. Cognitive behavioral therapy for fibromyalgia. Nat Clin Pract Rheumatol. (2006) 2:416–24. 10.1038/ncprheum0245 16932733

[53] Wetherell JL Afari N Rutledge T Sorrell JT Stoddard JA Petkus AJ. A randomized, controlled trial of acceptance and commitment therapy and cognitive-behavioral therapy for chronic pain. Pain. (2011) 152:2098–107. 10.1016/j.pain.2011.05.016 21683527

[54] Duay DL Bryan VC. Senior adults' perceptions of successful aging. Educ Gerontol. (2006) 32:423–45. 10.1080/03601270600685636

[55] Reid MC Otis J Barry LC Kerns RD. Cognitive–behavioral therapy for chronic low back pain in older persons: a preliminary study. Pain Med. (2003) 4:223–30. 10.1046/j.1526-4637.2003.03030.x 12974821

[56] Nielsen SS Christensen JR Søndergaard J Mogensen VO Enemark Larsen A Skou ST. Feasibility assessment of an occupational therapy lifestyle intervention added to multidisciplinary chronic pain treatment at a Danish pain centre: a qualitative evaluation from the perspectives of patients and clinicians. Int J Qual Stud Health Well-being. (2021) 16:1949900. 10.1080/17482631.2021.1949900 34252015 PMC8276665

[57] Flynn T-J Gow AJ. Examining associations between sexual behaviours and quality of life in older adults. Age Ageing. (2015) 44:823–8. 10.1093/ageing/afv083 26178206

[58] Agochukwu-Mmonu N Malani PN Wittmann D Kirch M Kullgren J Singer D. Interest in sex and conversations about sexual health with health care providers among older US adults. Clin Gerontol. (2021) 44:299–306. 10.1080/07317115.2021.1882637 33616005

[59] Smith LJ Mulhall JP Deveci S Monaghan N Reid M. Sex after seventy: a pilot study of sexual function in older persons. J Sex Med. (2007) 4:1247–53. 10.1111/j.1743-6109.2007.00568.x 17727349

[60] Lamé IE Peters ML Vlaeyen JW. Kleef Mv, Patijn J. Quality of life in chronic pain is more associated with beliefs about pain, than with pain intensity. Eur J Pain. (2005) 9:15–24. 10.1016/j.ejpain.2004.02.006 15629870

[61] Darnall BD Roy A Chen AL Ziadni MS Keane RT You DS. Comparison of a single-session pain management skills intervention with a single-session health education intervention and 8 sessions of cognitive behavioral therapy in adults with chronic low back pain: a randomized clinical trial. JAMA Netw Open. (2021) 4:e2113401-e. 10.1001/jamanetworkopen.2021.13401 34398206 PMC8369357

[62] Watson JA Ryan CG Cooper L Ellington D Whittle R Lavender M. Pain neuroscience education for adults with chronic musculoskeletal pain: a mixed-methods systematic review and meta-analysis. J Pain. (2019) 20:1140. e1–22. 10.1016/j.jpain.2019.02.011 30831273

[63] Gilam G Sturgeon JA You DS Wasan AD Darnall BD Mackey SC. Negative affect–related factors have the strongest association with prescription opioid misuse in a cross-sectional cohort of patients with chronic pain. Pain Med. (2020) 21:e127–e38. 10.1093/pm/pnz249 31617916 PMC7049262

[64] Sharifzadeh Y Kao M-C Sturgeon JA Rico TJ Mackey S Darnall BD. Pain catastrophizing moderates relationships between pain intensity and opioid prescription: nonlinear sex differences revealed using a learning health system. Anesthesiology. (2017) 127:136–46. 10.1097/ALN.0000000000001656 28614083 PMC5478434

[65] Booker SQ Sibille KT Terry EL Cardoso JS Goodin BR Sotolongo A. Psychological predictors of perceived age and chronic pain impact in individuals with and without knee osteoarthritis. Clin J Pain. (2020) 36:569. 10.1097/AJP.0000000000000842 32398442 PMC7335325

[66] Wakefield EO Puhl RM Litt MD Zempsky WT. “If It Ever Really Hurts, I Try not to let them know:” the use of concealment as a coping strategy among adolescents with chronic pain. Front Psychol. (2021) 12:1840. 10.3389/fpsyg.2021.666275 34149560 PMC8209248

[67] Molton IR Hirsh AT Smith AE Jensen MP. Age and the role of restricted activities in adjustment to disability-related pain. J Health Psychol. (2014) 19:1025–34. 10.1177/1359105313483156 23720543

[68] Cook KF Bamer AM Amtmann D Molton IR Jensen MP. Six patient-reported outcome measurement information system short form measures have negligible age-or diagnosis-related differential item functioning in individuals with disabilities. Arch Phys Med Rehabil. (2012) 93:1289–91. 10.1016/j.apmr.2011.11.022 22386213

